# Download Your Doctor: Implementation of a Digitally Mediated Personal Physician Presence to Enhance Patient Engagement With a Health-Promoting Internet Application

**DOI:** 10.2196/resprot.5232

**Published:** 2016-03-04

**Authors:** Charilaos Lygidakis, Paul Wallace, Costanza Tersar, Francesco Marcatto, Donatella Ferrante, Roberto Della Vedova, Francesca Scafuri, Emanuele Scafato, Pierluigi Struzzo

**Affiliations:** ^1^ Centro Studi e Ricerche in Medicina Generale (CSeRMEG) Monza Italy; ^2^ Department of Primary Care and Population Health University College London London United Kingdom; ^3^ Department of Life Sciences University of Trieste Trieste Italy; ^4^ Region Friuli Venezia Giulia Regional Centre for the Training in Primary Care Monfalcone Italy; ^5^ Istituto Superiore di Sanità WHO Collaborating Centre for Research and Health Promotion on Alcohol and Alcohol-Related Health Problems, Osservatorio Nazionale Alcol Centro Nazionale di Epidemiologia, Sorveglianza e Promozione della Salute Rome Italy

**Keywords:** alcohol drinking, physician-patient relations, behavior and behavior mechanisms, research design, Internet, multimedia, primary health care, family physicians

## Abstract

**Background:**

Brief interventions delivered in primary health care are effective in reducing excessive drinking; online behavior-changing technique interventions may be helpful. Physicians may actively encourage the use of such interventions by helping patients access selected websites (a process known as “facilitated access”). Although the therapeutic working alliance plays a significant role in the achievement of positive outcomes in face-to-face psychotherapy and its development has been shown to be feasible online, little research has been done on its impact on brief interventions. Strengthening patients’ perception of their physician’s endorsement of a website could facilitate the development of an effective alliance between the patient and the app.

**Objective:**

We describe the implementation of a digitally mediated personal physician presence to enhance patient engagement with an alcohol-reduction website as part of the experimental online intervention in a noninferiority randomized controlled trial. We also report the feedback of the users on the module.

**Methods:**

The Download Your Doctor module was created to simulate the personal physician presence for an alcohol-reduction website that was developed for the EFAR-FVG trial conducted in the Italian region of Friuli-Venezia-Giulia. The module was designed to enhance therapeutic alliance and thus improve outcomes in the intervention group (facilitated access to the website). Participating general and family practitioners could customize messages and visual elements and upload a personal photo, signature, and video recordings. To assess the perceptions and attitudes of the physicians, a semistructured interview was carried out 3 months after the start of the trial. Participating patients were invited to respond to a short online questionnaire 12 months following recruitment to investigate their evaluation of their online experiences.

**Results:**

Nearly three-quarters (23/32, 72%) of the physicians interviewed chose to customize the contents of the interaction with their patients using the provided features and acknowledged the ease of use of the online tools. The majority of physicians (21/32, 57%) customized at least the introductory photo and video. Barriers to usage among those who did not customize the contents were time restrictions, privacy concerns, difficulties in using the tools, and considering the approach not useful. Over half (341/620, 55.0%) of participating patients completed the optional questionnaire. Many of them (240/341, 70.4%) recalled having noticed the personalized elements of their physicians, and the majority of those (208/240, 86.7%) reacted positively, considering the personalization to be of either high or the highest importance.

**Conclusions:**

The use of a digitally mediated personal physician presence online was both feasible and welcomed by both patients and physicians. Training of the physicians seems to be a key factor in addressing perceived barriers to usage. Further research is recommended to study the mechanisms behind this approach and its impact.

**Trial Registration:**

Clinicaltrials.gov NCT 01638338; https://clinicaltrials.gov/ct2/show/NCT01638338 (Archived by WebCite at http://www.webcitation.org/6f0JLZMtq)

## Introduction

Alcohol is the third leading cause of diseases and premature death globally [1], and it has been estimated that it accounts for 3.8% of deaths and 4.6% of disability-adjusted life years [2]. Brief interventions delivered in primary health care settings have been shown to be both efficacious and effective in reducing excessive drinking in Italy and worldwide [3-7]. However, some individual large-scale pragmatic trials have failed to report significant findings [8-10], and no reductions in drinking or very small effects were observed in results from studies in probation offices, emergency departments, and among college students in Sweden and New Zealand [11-14]. Remarkably, the optimal contents and delivery strategies and the impact of the brief interventions on alcohol problems and specific population groups are not clear [7]. Comparing different brief intervention programs may be challenging because of differences in their contexts, the implementation strategies, and the indicators employed [6]. Moreover, there are important barriers to implementation, such as insufficient training, lack of resources, and time constraints, which prevent brief interventions from being employed widely [4,15-17].

Internet interventions based on behavior change techniques may be helpful to tackle these barriers [4,18]. Health care professionals may actively encourage the use of such digital applications by helping patients access selected websites (this process is known as “facilitated access”) [19]. Initially adopted primarily for the management of patients with mental health problems including depression and anxiety [20], facilitated access has been extended to addictive behaviors including smoking cessation and alcohol screening, as well health promotion and the management of long-term conditions [4,21-25]. Ensuring effective patient interaction with the website following facilitated access may be an issue [26], and additional mechanisms are therefore likely to be necessary to achieve more consistent engagement. In this paper, we describe the development and use of digitally mediated personal physician presence to enhance patient engagement with an alcohol-reduction website as part of the experimental online intervention in a noninferiority randomized controlled trial [27]. The intervention was designed to reinforce patients’ perception of their physician’s endorsement of the website and thus promote the development of an effective therapeutic working alliance between the patient and the application.

Described in a seminal paper by Bordin [28], the therapeutic working alliance refers to the personal relationship between the therapist and patient that stems from a collaborative endeavor to attain the goals of the treatment. Such an agreement on goals and tasks and the establishment of a personal bond of reciprocal positive feelings constitute the cornerstones of the alliance. There is a large body of evidence suggesting that it plays an important role in the achievement of positive outcomes in face-to-face psychotherapy [29-31], where patients who experience a strong alliance show more motivation and invest more effort to complete their treatment [30]. The importance of the alliance is also highlighted by researchers who suggest that nonspecific factors are largely responsible for the outcomes of different psychotherapies; these factors are common in the majority of the therapeutic interventions and include the healing setting, the expectations of improvement, and the therapeutic bond [32]. Little research has been done on the role of the therapeutic working alliance in treatment of alcohol problems or in brief interventions, but evidence is beginning to emerge that it may have an important influence on patient outcomes [33].

Researchers argue that the quality of the therapeutic working alliance is largely determined by the therapist variation, which may also explain the therapist’s effects on the outcomes [32]. Patient expectations of Internet-delivered behavioral change techniques may play a significant role as well [31], and according to Jasper et al, the development of the therapeutic alliance online may require more time than when therapy is delivered face-to-face [34]. In the context of facilitated access, agreement on goals and tasks can generally be achieved relatively easily online through the offer of a menu of options [34,35], but the maintenance and enhancement of the therapeutic alliance is considerably more problematic.

In health-promoting websites selected for facilitated access, customization of the information delivered to patients combined with multimedia emulation of their physician’s presence online may increase patient engagement, potentially by strengthening the online therapeutic alliance. Based on this hypothesis, we developed the digitally mediated primary care physician presence (Download Your Doctor) as a mechanism for achieving this end. We describe below how this was implemented in the context of a trial designed to determine whether facilitated access by primary care physicians to an alcohol-reduction website was superior to traditional face-to-face brief intervention [27] and to report the feedback of the users on the module.

## Methods

The Download Your Doctor module was developed as an additional feature to an alcohol-reduction website [36] with the objective of generating a simulated personal physician presence in the online environment. The website was developed as part of a randomized controlled trial conducted in primary health care settings in the Italian Region of Friuli-Venezia-Giulia (EFAR-FVG), aiming to test the effectiveness of facilitated access to an alcohol-reduction website compared with standard face-to-face brief intervention [27,36]. Its contents and design were based on the UK counterpart, DownYourDrink*,* which has been described elsewhere [37,38]. The rationale for the trial is that general practitioners (GPs)/family physicians (FPs) are generally reluctant to screen for alcohol misuse and delivery rates of brief interventions are low [27,39]. The use of facilitated access to a selected website to provide patients with an opportunity to undertake screening and brief intervention online rather than in the consultation offers significant promise, but a trial was needed to establish noninferiority of this approach. The study protocol was approved by the Isontina Independent Local Health Unit Ethics Committee on June 14, 2012.

For the duration of the trial, the participating GPs/FPs provided facilitated access consisting of a short discussion followed by presentation to the patient of a pamphlet describing the website and containing the patient’s personal login code. The first 3 digits of the login code were specific to the general practitioner and the last 4 to the patient. The online alcohol screening module was based on the 3-item Alcohol Use Disorders Identification Test (AUDIT-C). Those scoring above the designated cut point were requested to provide consent to the trial and then undergo an online baseline assessment, followed by the randomized assignment to receive either online facilitated access to the alcohol-reduction website or the face-to-face intervention by their GP/FP. Follow-up assessment took place online at 3 and 12 months using the full version of the AUDIT questionnaire [40] and the EQ-5D [41].

**Figure 1:**
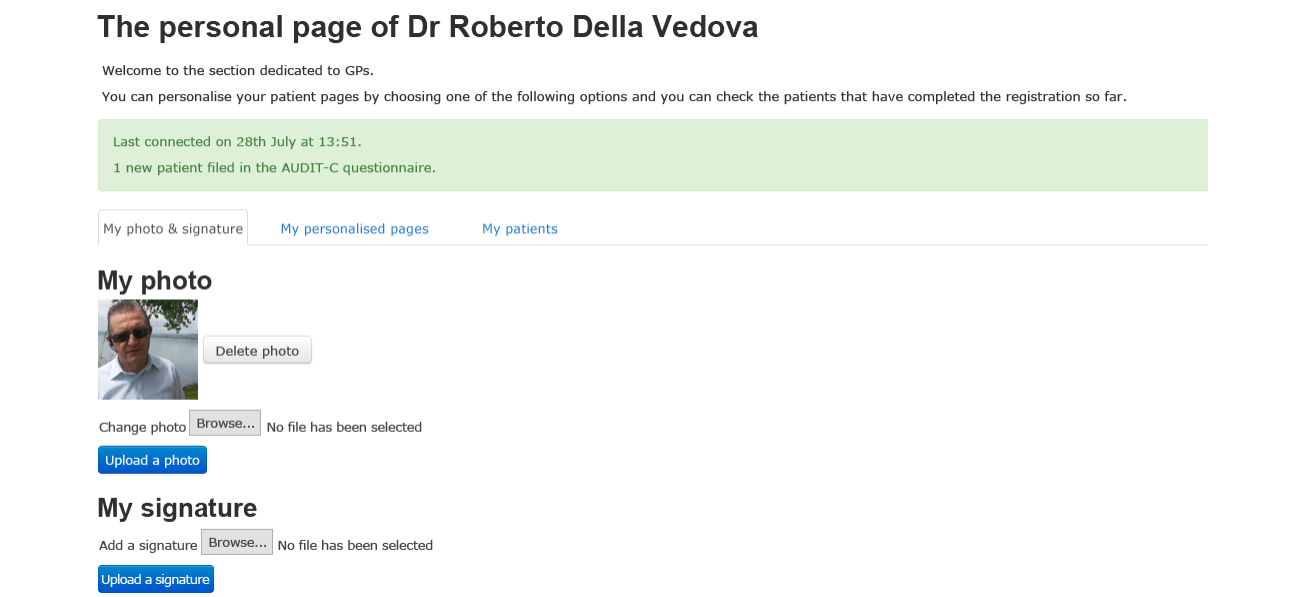
The GP/FP customization page (adapted in English).

**Figure 2:**
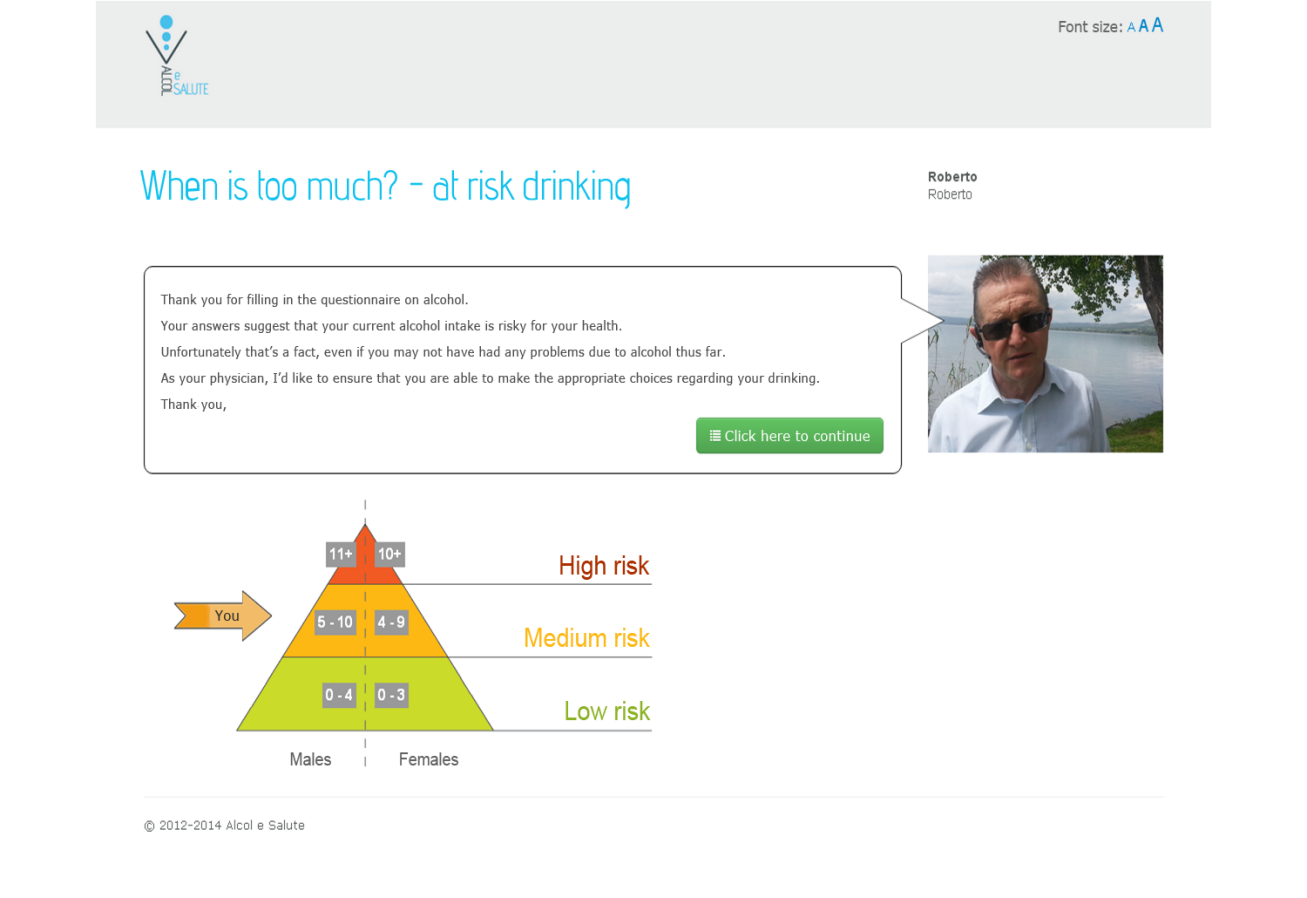
The screening page with the personalized speech bubble (adapted in English).

**Figure 3:**
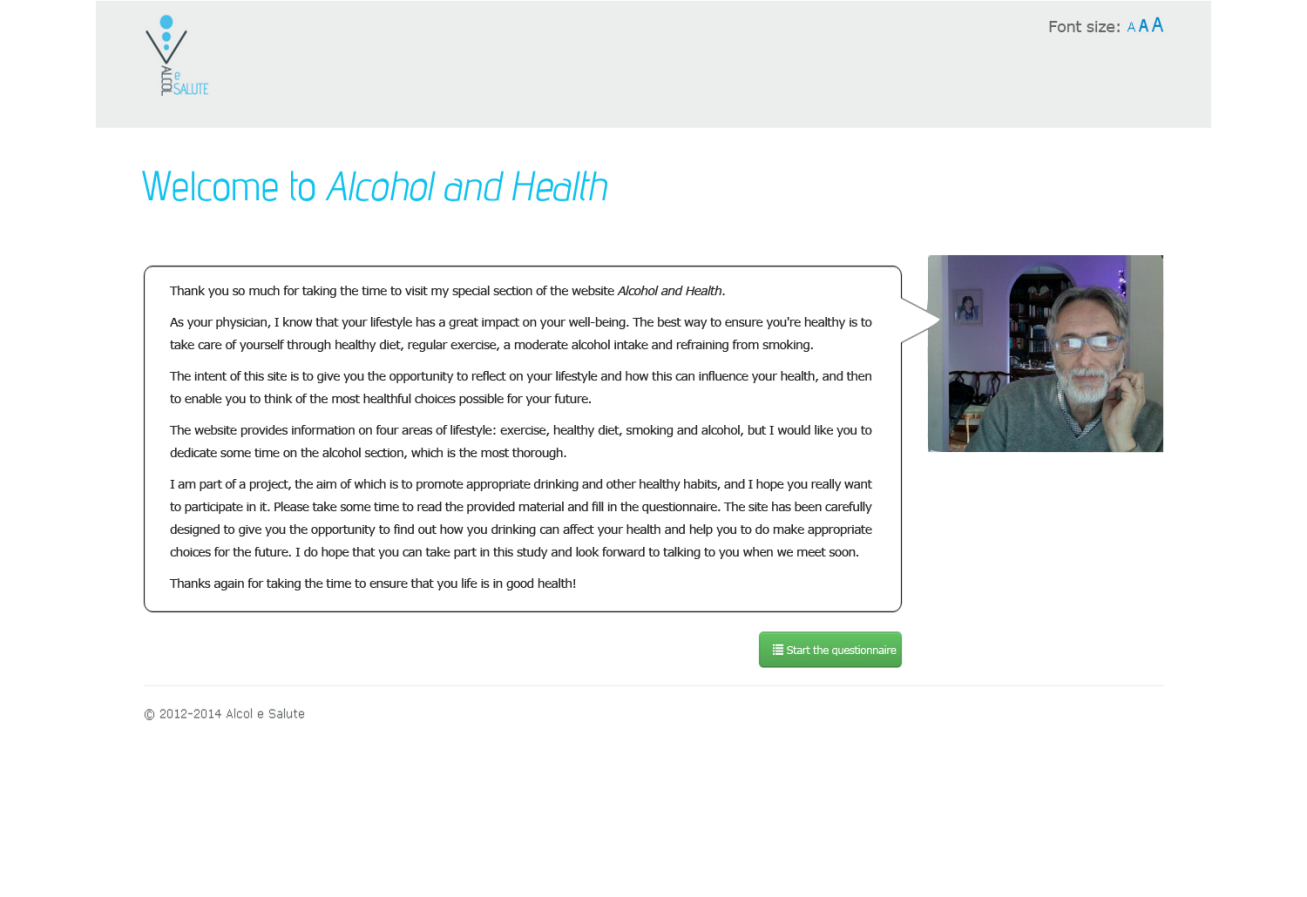
The personalized welcome page (adapted in English).

**Figure 4:**
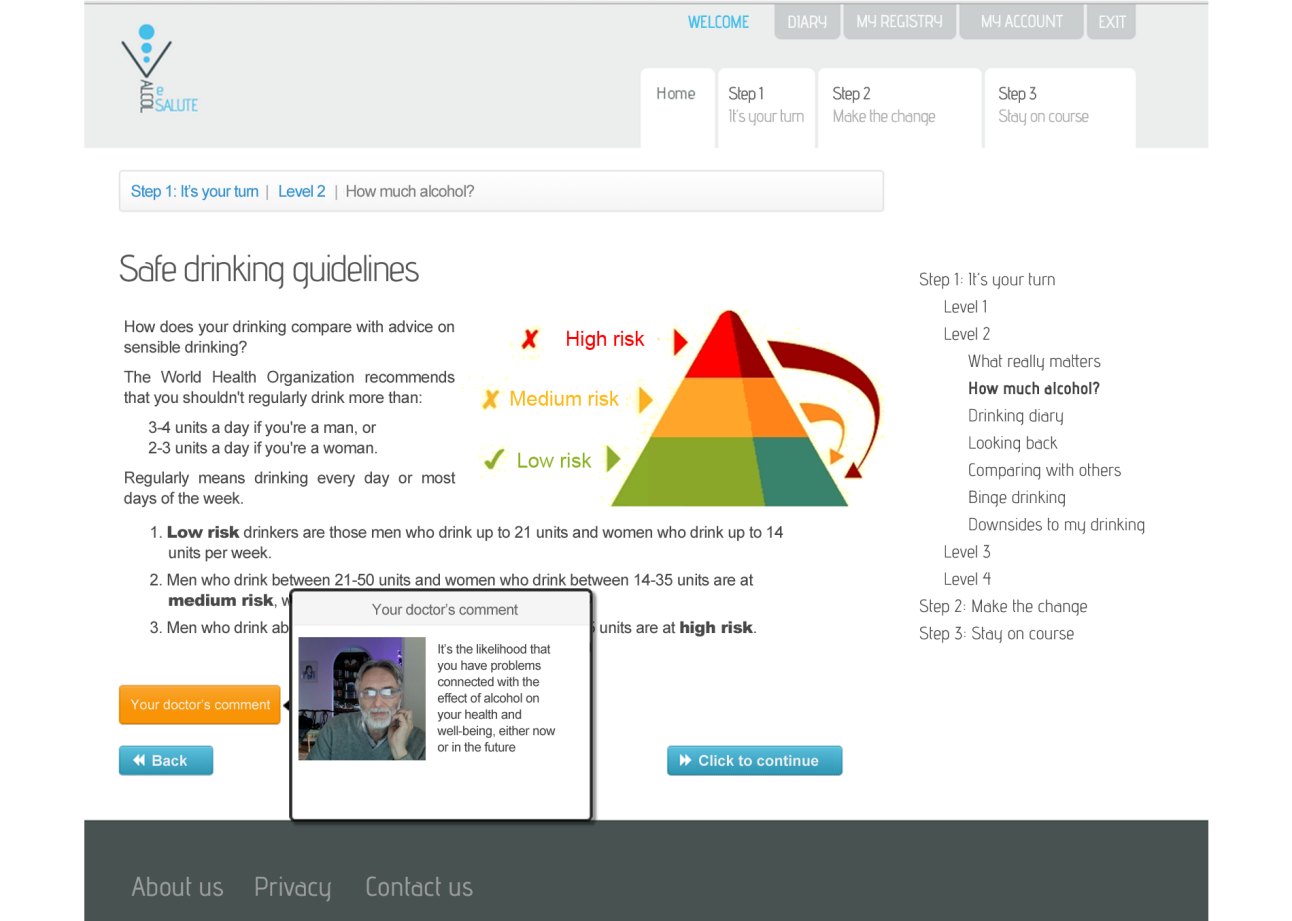
The GP/FP can provide customized comments on the different patient inputs (adapted in English).

The Download Your Doctor facility enabled the GPs/FPs participating in the study to personally customize selected messages and visual elements of the user interface to be presented to their patients after logging on to the website and following screening (see Figure 1). Each practitioner was additionally able to upload their personal photograph and signature for integration with their messages on the website. The appearance of each message could thus be customized by inserting links and images shown in a “speech bubble” next to text and along with the photo of the GP/FP (see Figures 2-4). Finally, they were also given the option to upload video recordings in order to simulate online communication with their patients even more directly.

Before the onset of the trial, the participating GPs/FPs were invited to attend a training session, during which a stepwise presentation of the website was carried out and the Download Your Doctor module was explained with interactive examples. Particular attention was given to the ways the physicians could enhance patient participation and adherence to the study. Subsequent use of the module by each participating physician was recorded by the study team.

To assess the perceptions and the attitudes of the physicians with regard to their participation in the study, an interview was conducted 3 months after the start of the trial. The interview was conducted by 2 psychologists using a semistructured questionnaire format. It sought information on such aspects of the trial as the nature of physicians’ interactions with the patients (recruitment and instructions) and their satisfaction with the participation in the trial. During the interview, the use and evaluation of the website (personalization, usability, and usefulness of the content) were also explored.

Moreover, a short online questionnaire was developed post hoc by the research team to evaluate the perceptions and attitudes of the trial participants about their experience in the study, their use of Internet and health-related websites, and the quality of their online experiences. A number of questions specifically sought information about the patient perceptions of the importance of the features related to the Download Your Doctor module. Five categories were available to rate the features (“highest,” “high,” “average,” “low,” “lowest”). Completion of the questionnaire was optional, and it was included in the final assessment that each patient was requested to undertake online 12 months following recruitment to the trial.

## Results

Of the 44 participating GP/FPs approached by the research team, 32 (73%) agreed to be interviewed, and 23 (72%) of those used the Download Your Doctor module and personalized some of its parts by uploading photos and/or adding their own text messages (see Table 1).

**Table 1:** Personalization of the website (and lack of) among interviewed doctors (N=32).

Personalization details		n (%)
**Interviewed doctors who personalized one or more elements of the website**		23 (72)
	The introductory photo and video	21 (57)
	The introductory text	11 (34)
**Interviewed doctors’ reasons for not personalizing the website**		9 (28)
	Limited time	3 (33)
	Seemed to be complicated	2 (22)
	Seemed not useful	2 (22)
	Unwilling to publish personal photos on the Internet	3 (33)

### Physicians’ Evaluation of the Website

According to the feedback from the physicians, the website scored highly in the aspects of user-friendliness and intuitive navigation, while overall the contents were considered to be useful for their patients (see Table 2). No significant differences emerged between physicians who personalized the website and those who did not do so with regard to the aforementioned aspects of user-friendliness and content usefulness, as well as the level of interest and overall satisfaction with the participation in the trial (*P*>.10).

**Table 2:** Physician feedback on the website based on Likert scales, ranging from 1-10 (lowest-highest).

Survey question	Median	Interquartile range
To what degree do you find the website easy to browse and interact with?	10	8-10
To what extent do you rate the website’s contents and activities to be useful for the patients?	8	8-10

### Patients’ Evaluation of the Website

Of the 620 patients who completed the 12-month follow-up questionnaires, 341 (55.0%) also completed the optional questionnaire. The demographic characteristics and information technology (IT) skills of the sample are shown in Table 3.

**Table 3:** Demographic characteristics and IT skills of the sample (N=341; only valid answers are shown).

Characteristics		Values
**Gender, n (%)**		
	Males	215 (64.2)
	Females	120 (35.8)
	Age, mean (SD) interquartile range	47.27 (14.60) 36.00-58.00
**IT skills, n (%)**		
	Poor	47 (14.0)
	Basic	90 (26.9)
	Intermediate	82 (24.5)
	Advanced	116 (34.6)

Nearly three-quarters (240/341, 70.4%) of the patients recalled having noticed the personalized elements of their GPs/ FPs (see Table 4). There were no significant differences in the gender, age, and IT skills between the participants who noticed the personalization and those who did not (*P*≥.05). Similarly, the two groups did not differ in the frequency with which the participants used the Internet and various health-related websites (*P*≥.05).

Of those patients who noticed the personalization, the majority (208/240, 86.7%) considered such an approach to be of either high or the highest importance for their level of confidence towards the information provided by the website (see Table 4). There were no significant correlations between the level of their confidence and the age, IT skills, and frequency with which the Internet and the health-related websites were employed (*P*≥.05), while the level of their confidence did not differ between the two sexes (*P*≥.05).

Furthermore, approximately half of those who did not notice any personalized element (55/101, 54.4%) considered such a feature to be potentially of either high or the highest importance (see Table 4).

**Table 4:** Patient feedback on website (N=341).

Questions		n (%)
**All respondents (n=341): “When you first logged into the website, do you remember having seen a photo or a text message from your GP?”**		
	Yes	240 (70.4)
	No	101 (29.6)
**Respondents who recalled GP personalization of the website (n=240): “To what extent do you think that seeing a photo or a message from your GP improved your confidence towards the information provided by the website?”**		
	Lowest	1 (0.4)
	Low	5 (2.1)
	Average	25 (10.5)
	High	74 (31.0)
	Highest	134 (56.1)
**Respondents who did not recall GP personalization of the website (n=101, valid answers=100): “To what extent do you think that seeing a photo or a message from your GP could have improved your confidence towards the information provided by the website?”**		
	Lowest	15 (14.9)
	Low	14 (13.9)
	Average	17 (16.8)
	High	37 (36.6)
	Highest	18 (17.8)

With regard to the participants’ evaluation of the website, only a few replies differed significantly between those who noticed the personalization and those who did not recall such elements (see Table 5). There were no significant differences between the two groups in the subjective evaluation of the individual alcohol consumption and the role that the website played (*P*≥.05): “Do you think that you have reduced your alcohol intake compared with a year ago?,” “Do you consider that your way of drinking today poses a risk to your health?,” “Do you think that the website was helpful in changing your way of drinking alcohol?”. Finally, the majority of the patients considered the website very easy to use, and no differences were found between the two groups (median 4.52 for those who recalled the personalization vs median 4.35 for those who did not recall such elements).

**Table 5:** Significant differences in the replies between the participants that recalled the personalization and those who did not do so. Answers were based on a 5-point Likert scale, ranging from 1-5 (lowest-highest).

Questions		N	Mean	Standard deviation	*t* (df)	*P*
“**Do you assess your participation in the study positively?”**						
	Respondents who recalled the personalization	238	4.09	0.86	3.18 (317)	.002
	Respondents who did not recall the personalization	101	3.74	1.04		
“**Did you find the contents of the site interesting?”**						
	Respondents who recalled the personalization	108	4.31	0.76	2.85 (154)	.005
	Respondents who did not recall the personalization	48	3.90	0.97		
“**Would you recommend the website to others?”**						
	Respondents who recalled the personalization	108	4.23	0.82	2.34 (154)	.020
	Respondents who did not recall the personalization	48	3.88	1.00		

## Discussion

### Principal Findings

The Download Your Doctor module was developed to enhance the therapeutic alliance between patients and physicians, in order to improve the outcomes of a trial on facilitated access to an alcohol-reduction website. Our hypothesis was that while patients may appreciate the opportunities presented through facilitated access, there might be a risk that they perceive it as less valuable than face-to-face intervention. It was therefore important to provide for continuity of the therapeutic alliance while the patient was online. The GPs/FPs were provided with features enabling them to customize the contents of the interaction with their patients and the majority of them chose to do so, acknowledging the ease of use of the online tools. Most patients reported noticing the novelty of this approach and reacted positively to such customizations.

Providing general health communication materials may have limited effect when the information is not relevant to the individual’s context [42]. Tailored communication has been used in behavioral interventions delivered in print and by telephone. Yet the advances of technology have yielded new possibilities for this increasingly common practice [43], and interventions using computer tailoring to assess individuals and deliver adapted content and feedback according to the user’s characteristics have been proven to result in more behavioral changes compared to standard programs [42,44,45]. Such interventions may be more effective as they contain fewer unnecessary messages and increase the relevance of the information to the individuals, who are more likely to assimilate it [43-48]. Although interventions using social networks seem to be effective in behavioral change and higher recruitment and retention may be achieved by taking advantage of the participants’ existing social networks [49], their implementation in alcohol-reduction applications seems to be scarce [18]. Furthermore, personalized feedback based on the patient’s answers may have a positive influence on the therapeutic working alliance in Internet-delivered programs [18,30] and there is some evidence that the use of multimedia emulating a human therapist may further enhance its strength [50,51]. Self-help texts may also help foster an alliance based on the assumption that the patient attributes the preparation of the text to an empathetic clinician thus considering him/her present during the program [35,52].

As with many digital health applications, barriers to usage may include time limitations, insufficient skills, and privacy concerns [53-55]. The level of digital literacy of the practitioners may have played a role in whether they customized the contents of the module or not. Even though digital literacy was not assessed, the feedback from physicians relating to navigation and interaction with the website was generally positive and suggested that it was feasible for the majority to carry out the tasks of personalization after the training session. In fact, training seems to be a key factor in addressing the perceived barriers to usage, and according to literature it can also tackle the time constraint concerns, as more physicians are able to recognize the potential usefulness of the proposed tool [55].

The study was exploratory in nature and has a number of limitations. Notwithstanding the patients attributing a high significance to the customization, its impact on the outcomes of the trial could not be robustly evaluated due primarily to the variable degree of customization applied by the GPs/FPs. Not only did the content and multimedia elements vary among the participating physicians, but also some of them delayed customization until after the onset of the study. The main trial was designed to measure the effectiveness of the facilitated access to a dedicated alcohol-reduction website compared with the standard face-to-face brief intervention in primary care settings, while the evaluation of the Download Your Doctor module was added post hoc. As a result, the methodology and the available data were not adequate to enable us to draw firm conclusions. For example, we were unable to exclude “false positive” cases, namely patients who stated that they had noticed customization elements when their doctors had not actually made any modifications to the original presentation of the website. Nonetheless, while the study should be considered exploratory, as far as we are aware it is the first to address the impact of a digitally mediated primary care physician presence in an online application. As such, we have reported primarily on the feasibility of the implementation of this approach, which provides a foundation for future research into improving the quality of online therapeutic alliances by offering continuity of the already established relationship between patient and physician in the context of facilitated access to an intervention website.

### Conclusion

We have described a novel approach using a digitally mediated primary care physician presence based on customized content by GPs/FPs in order to increase patient engagement and promote their online therapeutic working alliance. Our findings suggest that this approach was both feasible and welcomed by both patients and physicians. As behavior change interventions are based on multiple techniques that may work synergistically and have variable effects in different patient groups [18], further investigation should focus on determining the mechanisms of this approach and its impact on the outcomes facilitated access to Internet-delivered brief interventions programs.

## Abbreviations

AUDIT: Alcohol Use Disorders Identification Test
EFAR-FVG: effectiveness of primary care based Facilitated access to Alcohol Reduction website – a randomized controlled noninferiority trial in Region Friuli Venezia Giulia, IT
GP: general practitioner
FP: family physician
IT: information technology
